# Dataset on comparable magnetocaloric properties of melt-extracted Gd_36_Tb_20_Co_20_Al_24_ metallic glass microwires

**DOI:** 10.1016/j.dib.2019.104960

**Published:** 2019-12-07

**Authors:** Hangboce Yin, Yongjiang Huang, Ying Bao, Sida Jiang, Peng Xue, Songshan Jiang, Huan Wang, Faxiang Qin, Ze Li, Shuchao Sun, Yunfei Wang, Hongxian Shen, Jianfei Sun

**Affiliations:** a School of Materials Science and Engineering, Harbin Institute of Technology, Harbin 150001, China; b Institute for Composites Science Innovation, School of Materials Science and Engineering, Zhejiang University, Hangzhou, 310027, China

**Keywords:** Metallic glass, Microwires, Magnetic refrigeration, Magnetocaloric effect

## Abstract

The data in this article is the supplementary data of the research article entitled “Comparable magnetocaloric properties of melt-extracted Gd_36_Tb_20_Co_20_Al_24_ metallic glass microwires” (Yin et al., 2020). The data shows the circular cross section of Gd_36_Tb_20_Co_20_Al_24_ metallic glass microwires with a diameter of ∼55 μm. The data also shows that the chemical compositions of microwires are basically uniform on macro-scale and micro-scale.

**Table:** Specifications Table

Subject	Physics
Specific subject area	Materials Science
Type of data	Image
How data were acquired	Scanning electron microscopy (SEM, FEI Quanta 200FEG) equipped with an energy dispersive X-ray spectroscopy (EDS), scanning transmission electron microscopy (S/TEM, FEI Talos F200X) equipped with an energy dispersive X-ray spectroscopy (EDS)
Data format	Raw
Parameters for data collection	The parameters of SEM and EDS are 2500 x of magnification, 20 kV of high voltage and 14.2 mm of work distance. The parameters of S/TEM and EDS are 225 kx of magnification, 200 kV of high voltage and 260 mm of work distance.
Description of data collection	The microstructure of Gd_36_Tb_20_Co_20_Al_24_ metallic glass microwires were observed by SEM and S/TEM, respectively. The compositions distributions of Gd, Tb, Co and Al elements were examined by SEM-EDS and S/TEM-EDS.
Data source location	Harbin, China
Data accessibility	The data are available with this article.
Related research article	Hangboce Yin, Yongjiang Huang, Ying Bao, Sida Jiang, Peng Xue, Songshan Jiang, Huan Wang, Faxiang Qin, Ze Li, Shuchao Sun, Yunfei Wang, Hongxian Shen, Jianfei Sun Comparable magnetocaloric properties of melt-extracted Gd_36_Tb_20_Co_20_Al_24_ metallic glass microwires Journal of Alloys and Compounds. 10.1016/j.jallcom.2019.06.085

**Table:** 

**Value of the Data** • This data fulfills the cross-section microstructure and the chemical compositions line distributions of Gd_36_Tb_20_Co_20_Al_24_ metallic glass microwires on macro-scale. • This data presents the microstructure and the chemical compositions distributions of microwires on micro-scale. • This data is useful in understanding the shape characteristics, microstructure and chemical compositions distributions of microwires.

## 1 Data

**Fig. 1:**
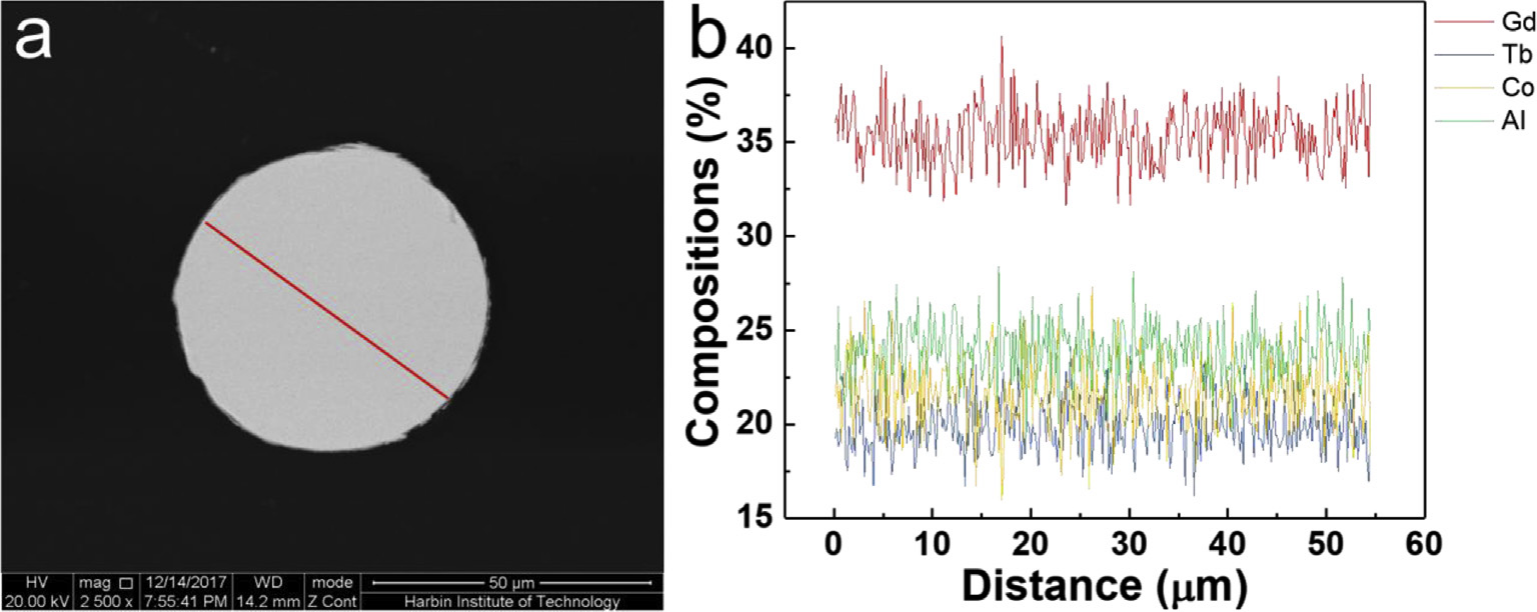
(a) SEM image of Gd_36_Tb_20_Co_20_Al_24_ metallic glass microwire showing the cross-section microstructure, (b) EDS line profiles of Gd, Tb, Co and Al elements in the radial direction as indicated by the red line in (a).

**Fig. 2:**
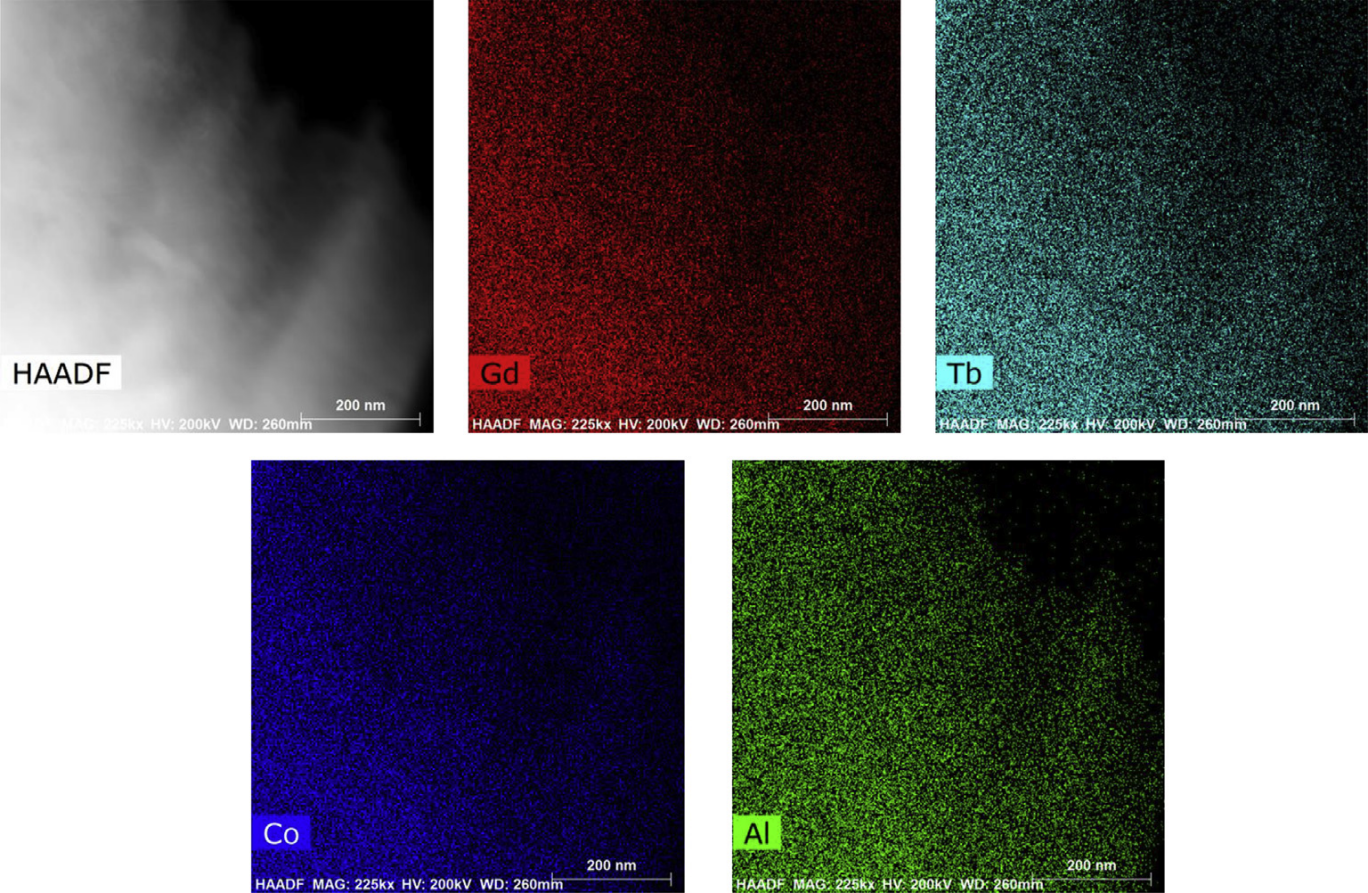
HAADF S/TEM image and EDS mapping results of Gd_36_Tb_20_Co_20_Al_24_ metallic glass microwires.

This dataset provides the information on the scanning electron microscopy (SEM) image, the high-angle annular dark-field (HAADF) scanning transmission electron microscopy (S/TEM) image and energy dispersive X-ray spectroscopy (EDS) results of Gd_36_Tb_20_Co_20_Al_24_ metallic glass microwires [1]. Fig. 1a shows the SEM image of cross-section microstructure of Gd_36_Tb_20_Co_20_Al_24_ metallic glass microwires. Fig. 1b presents the EDS line profiles of Gd, Tb, Co and Al elements in the radial direction as indicated by the red line in Fig. 1a. The HAADF S/TEM image and EDS mapping results of microwires are shown in Fig. 2.

## 2 Experimental design, materials, and methods

The preparation process of Gd_36_Tb_20_Co_20_Al_24_ metallic glass microwires was described in Ref. [2]. RE_36_RE_20_Co_20_Al_24_ alloy ingots with a nominal composition of Gd_36_Tb_20_Co_20_Al_24_ were prepared by arc melting a mixture of Gd, Tb, Co and Al metals with the purities of higher than 99.9 wt% in a Ti-gettered high-purity Ar atmosphere, followed by drop casting the melt into a copper mold. The dimensions of the obtained cylindrical samples were 10 mm in diameter and 70 mm in length. Subsequently, the rod was placed in a boron-nitride crucible and melted by a high frequency induction furnace. Finally, the melt was extracted by a molybdenum wheel, 60 knife-edge and 320 mm in diameter. The constant linear velocity of the wheel was 30 m/s and the feeding rate of the melt was 15–30 μm/s. Fig. 1a shows the circular cross section of Gd_36_Tb_20_Co_20_Al_24_ metallic glass microwires with a diameter of ∼55 μm. Fig. 1b presents that in the radial direction, the chemical compositions of microwires are basically uniform and close to the nominal compositions. The EDS mapping results indicate that chemical compositions of microwires are evenly distributed on micro-scale without segregation, as shown in Fig. 2.
